# Long-Term Survival of Dental Implants in a Patient With Periodontitis and Diabetes Mellitus: An Eight-Year Follow-Up Case Report

**DOI:** 10.7759/cureus.75852

**Published:** 2024-12-17

**Authors:** Mayur Kaushik, Aprajita Srivastava, Shivi Khattri

**Affiliations:** 1 Department of Periodontology &amp; Implantology, Subharti Dental College and Hospital, Swami Vivekanand Subharti University, Meerut, IND

**Keywords:** dental implantology, dental implant survival, long term survival, periodontal disease (pd), type i diabetes mellitus

## Abstract

Dental implants are now a standard solution for replacing missing teeth, even in patients with a history of chronic periodontitis. India is often referred to as the "diabetic capital of the world," a title that reflects the country's alarming rates of diabetes prevalence. However, the risk of complications, such as peri-implantitis and implant failure, remains a concern for these patients. This clinical case investigates the long-term success and survival of dental implants in patients with chronic periodontitis and diabetes mellitus. Results from an eight-year follow-up suggest that with appropriate periodontal treatment, careful implant placement, and regular maintenance, dental implants can remain functional and stable, even in individuals with chronic periodontal disease and diabetes mellitus.

## Introduction

Periodontitis is a chronic inflammatory condition that leads to the progressive destruction of tooth-supporting structures, including the alveolar bone [1]. If left untreated, it can lead to mobility of teeth and subsequently tooth loss. The complications can be compounded by the presence of systemic diseases, e.g., diabetes mellitus (DM). DM, particularly when poorly controlled, has systemic effects on wound healing, immune function, and bone metabolism. Elevated blood glucose levels impair collagen formation, reduce neutrophil function, and delay bone healing [2].

Dental implants have become the gold standard for replacing missing teeth, offering a permanent, functional, and aesthetic solution. However, their long-term success can also be influenced by several factors, including underlying health conditions [3]. The presence of DM may affect osseointegration, the process by which the dental implant fuses with the bone [2]. Thus, the simultaneous presence of periodontitis and DM can cumulatively affect the implant success [4]. These patients are often at an increased risk for developing peri-implantitis following dental implant placement due to the potential transfer of periodontal pathogens to the implant site [5]. This further compromises the stability of dental implants [6]. This raises the question of whether implants should be considered in patients with a history of periodontal disease and DM.

Studies have shown that patients with a history of periodontitis and DM may be more susceptible to biological complications around implants, such as peri-implantitis and implant failure, when compared to healthy patients [7]. Despite these concerns, implant therapy has been shown to be successful in periodontally compromised as well as in diabetic patients, provided that adequate treatment and maintenance protocols are followed [8].

The aim of this case report is to present a patient with long-term survival of dental implants even when complexed by periodontitis and long-standing DM.

## Case presentation

**Figure 1:**
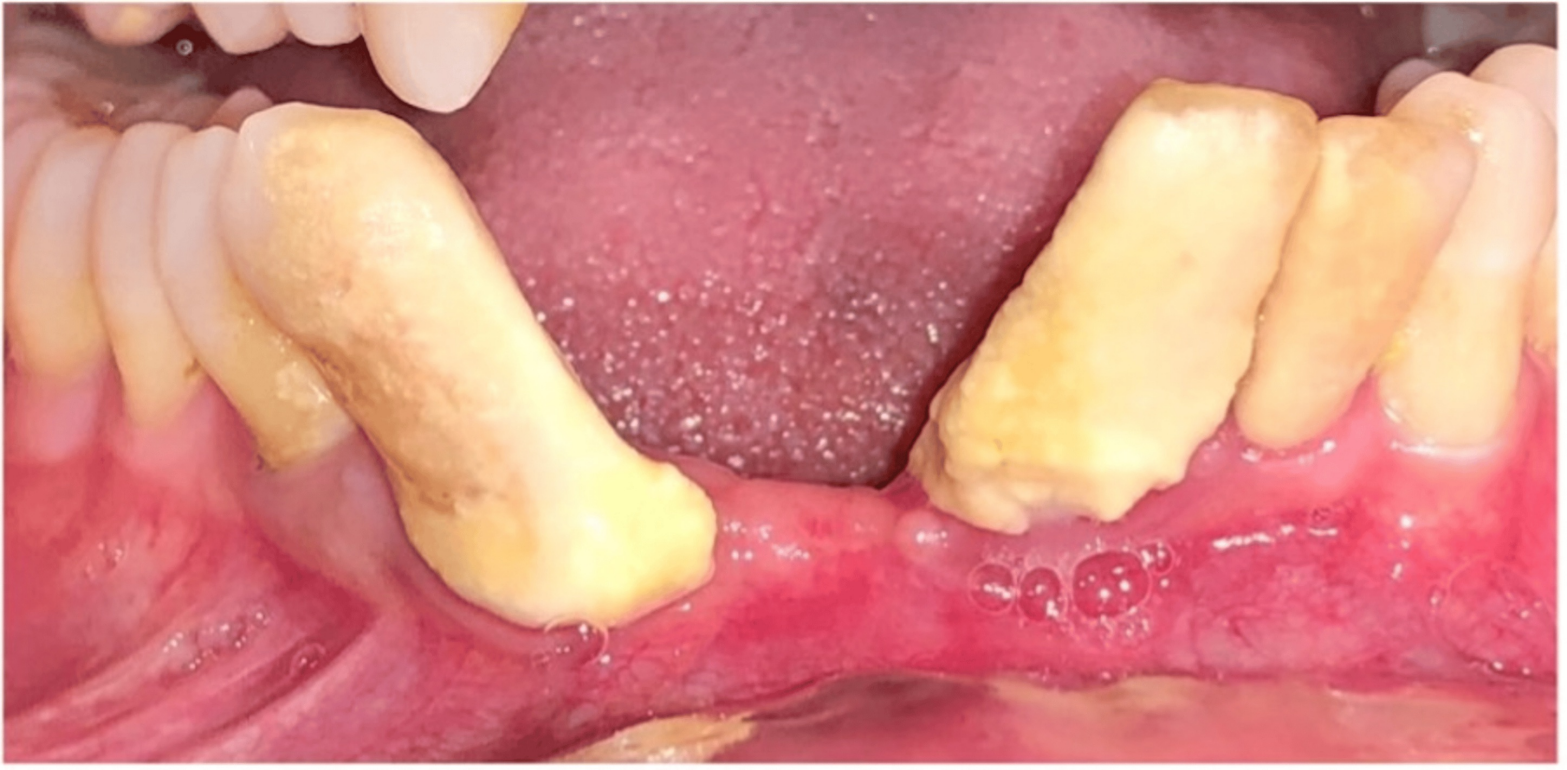
Pre-operative view

A 42-year-old male presented to the outpatient Department of Subharti Dental College and Hospital, Meerut, India, with a chief complaint of inability to chew food. He gave a history of type 2 DM for which he was on medication for the past 10 years. The patient's fasting blood glucose levels were 130mg/dL and HbA1c was 6.7. This indicates that the DM was controlled. On oral examination, there was generalized periodontitis (stage III, grade B). Severe attachment loss and grade III mobility was present on mandibular teeth #31 and #43. Teeth #41 and #42 were missing (Figure 1). The reason for poor oral hygiene was inadequate awareness and careless approach towards dental care.

Treatment planning

Meticulous phase I periodontal therapy comprising of scaling and root planing, oral hygiene instructions, and chlorhexidine mouthwash. Phase 2 periodontal therapy comprised of full mouth flap surgery. The extraction of the hopeless teeth and immediate implant placement were planned for the mandibular anterior region.

Surgical phase

After phase I therapy, informed consent was duly signed by the patient. At the follow-up visit, the gingival inflammation had subsided but the pockets still persisted. Full mouth flap surgery, i.e., open flap debridement using Kirkland flap design was performed except for the mandibular anterior region. The patient was put on the maintenance phase. After three months, the patient was deemed stable for oral hygiene maintenance. The patient was advised to undergo cone beam computed tomography. to determine the availability of the bone and pre-plan the angulation for implant placement.

**Figure 2:**
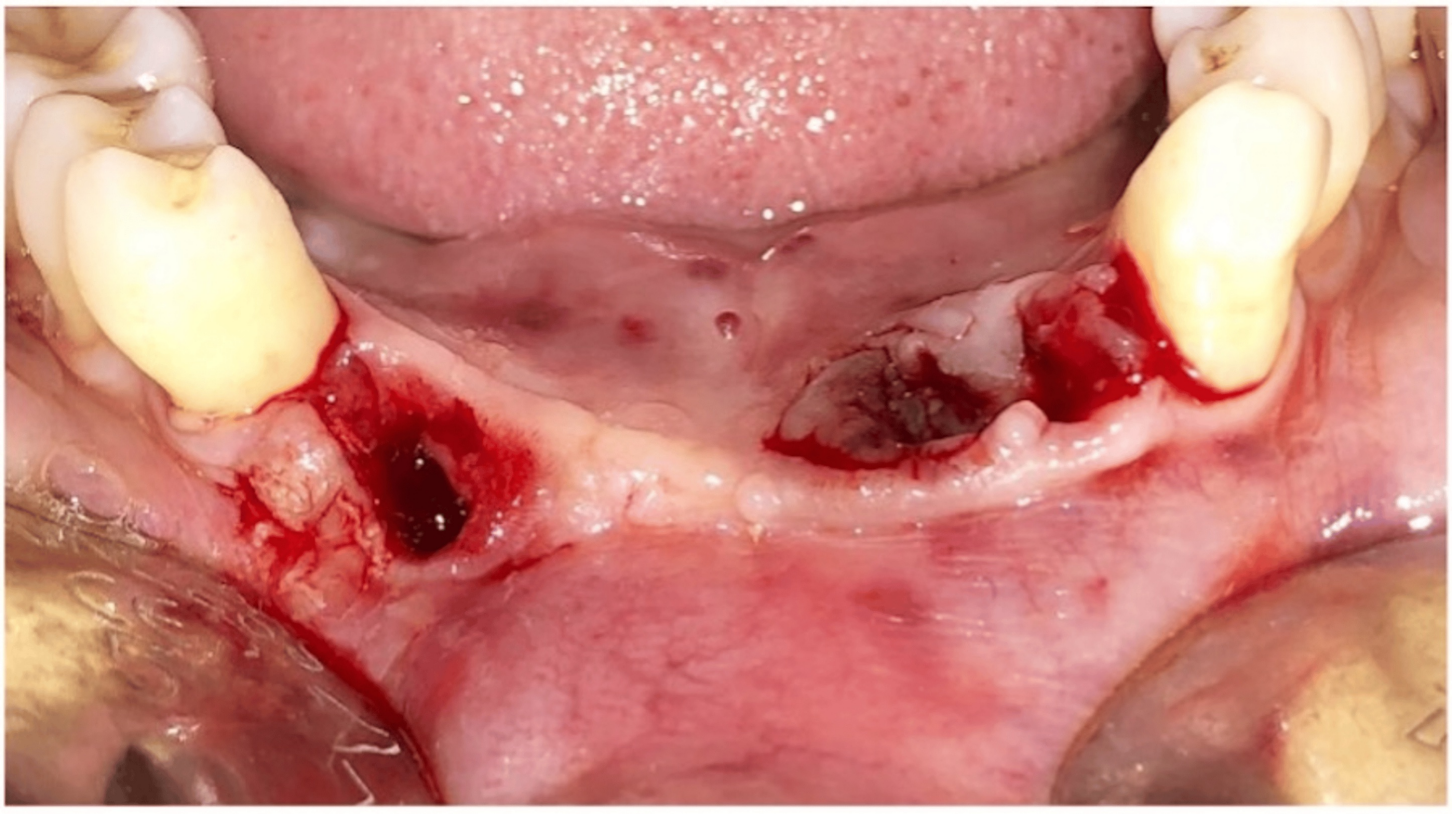
Teeth #43 and #31 extracted

**Figure 3:**
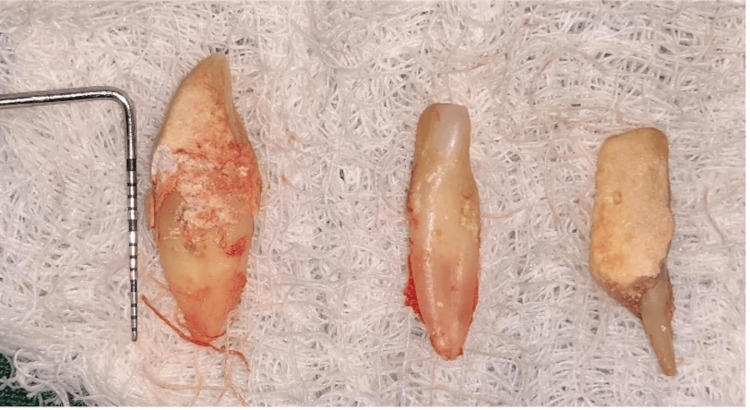
Extracted teeth

**Figure 4:**
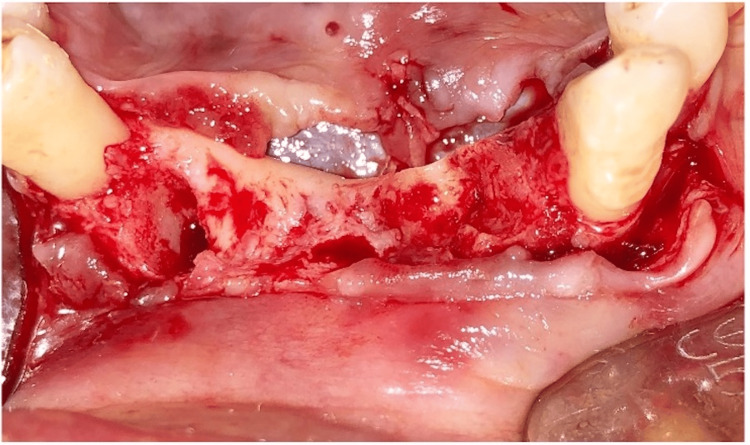
Flap reflection

**Figure 5:**
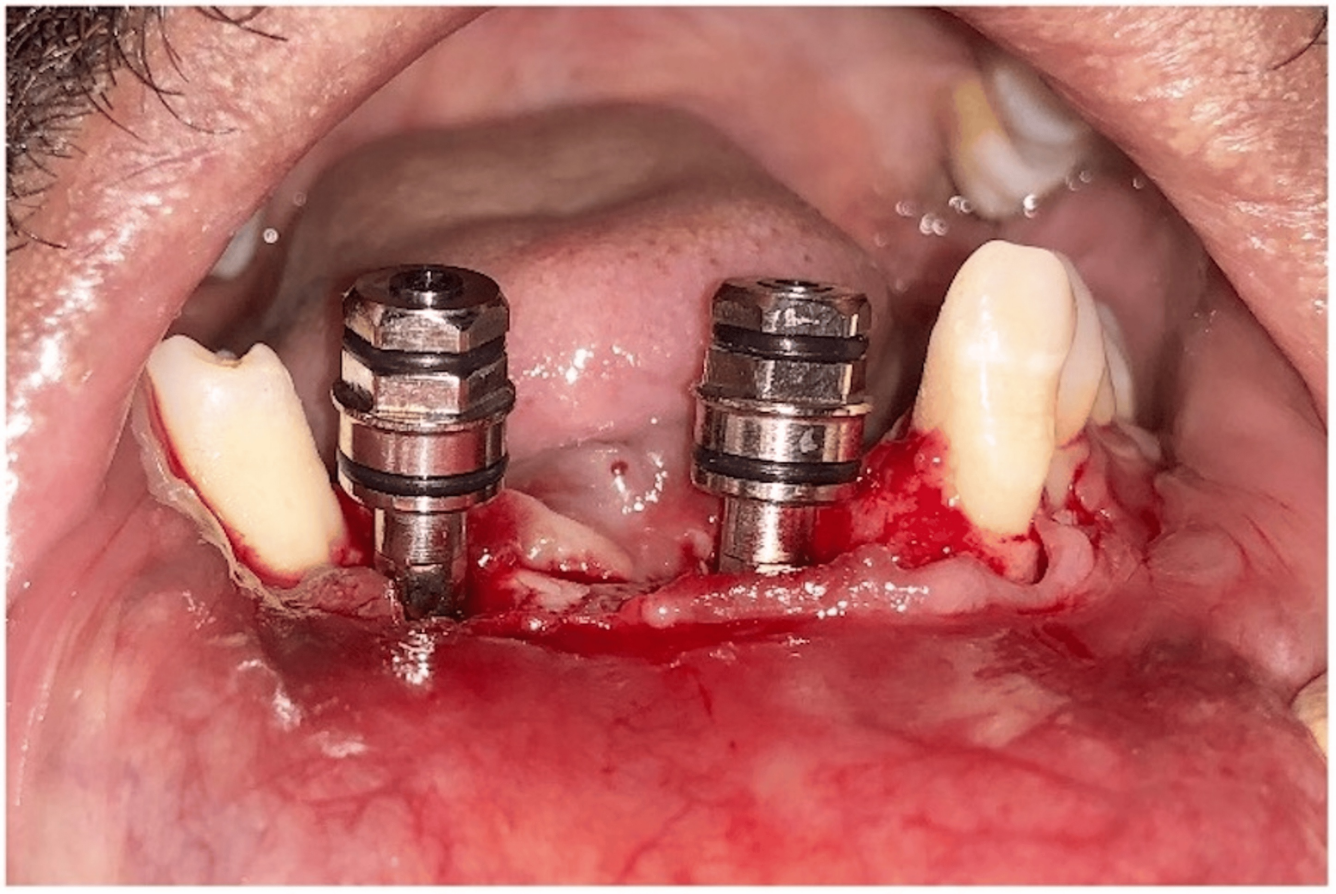
Implant placement

**Figure 6:**
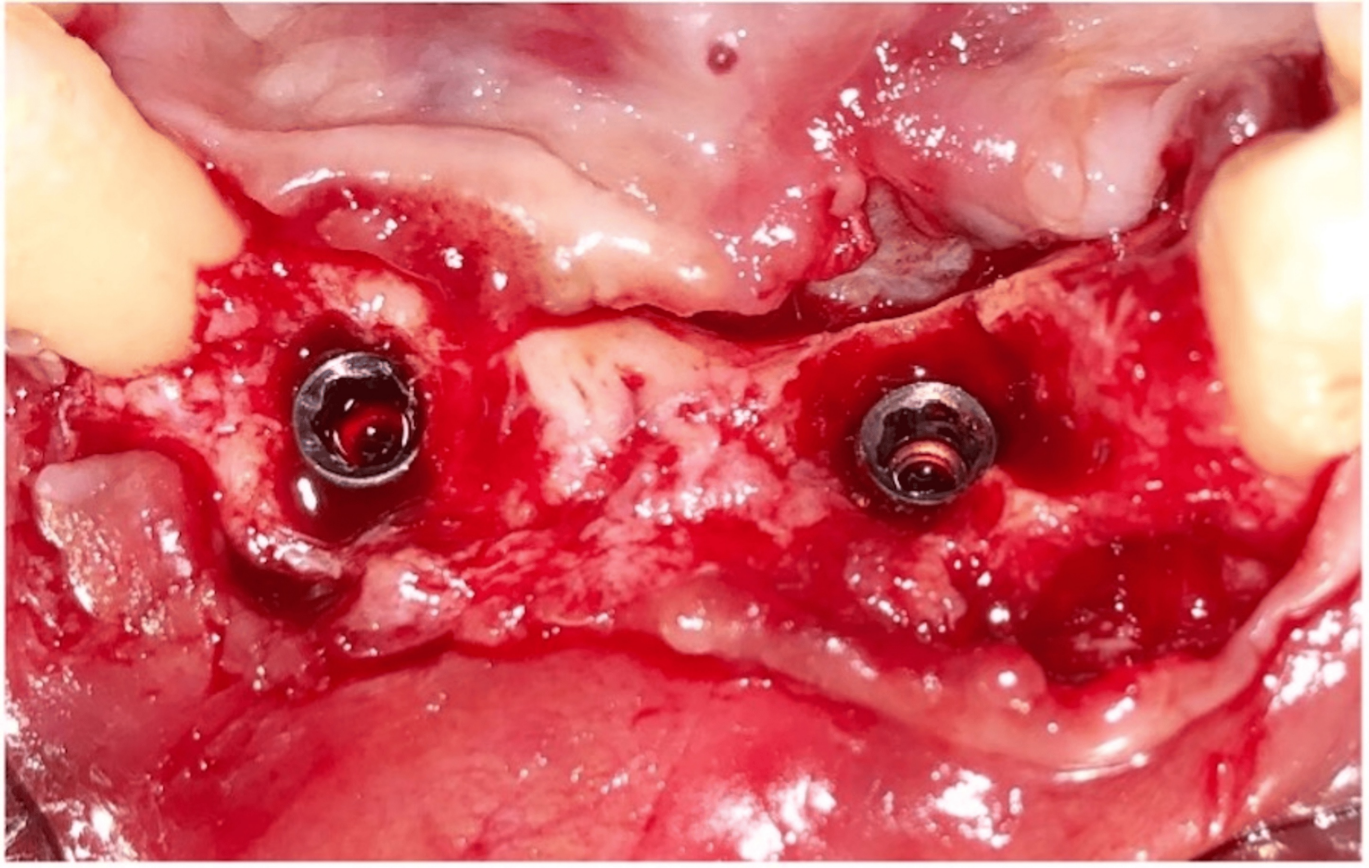
Sub-crestal placement of the implant

**Figure 7:**
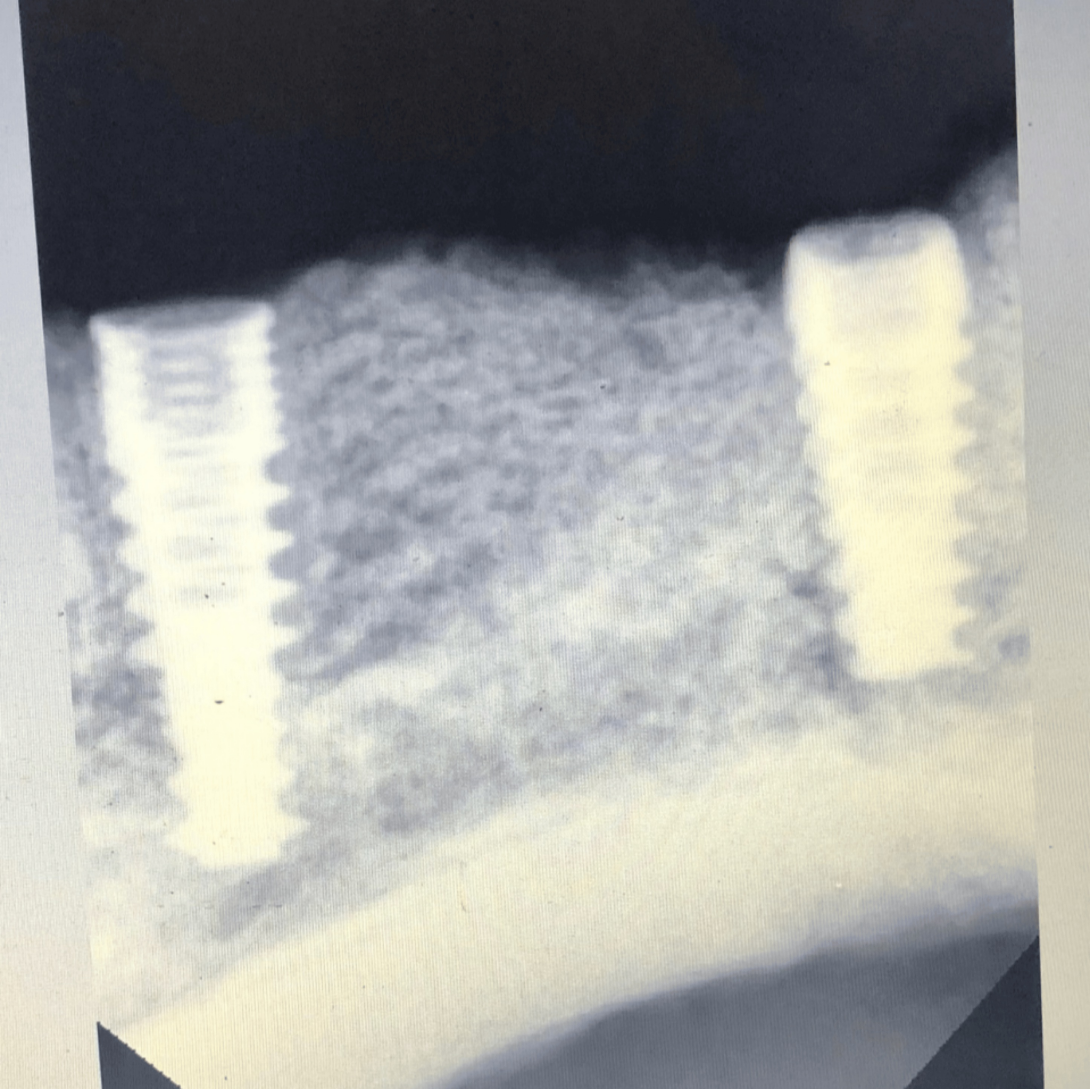
Radiograph immediately after implant placement.

**Figure 8:**
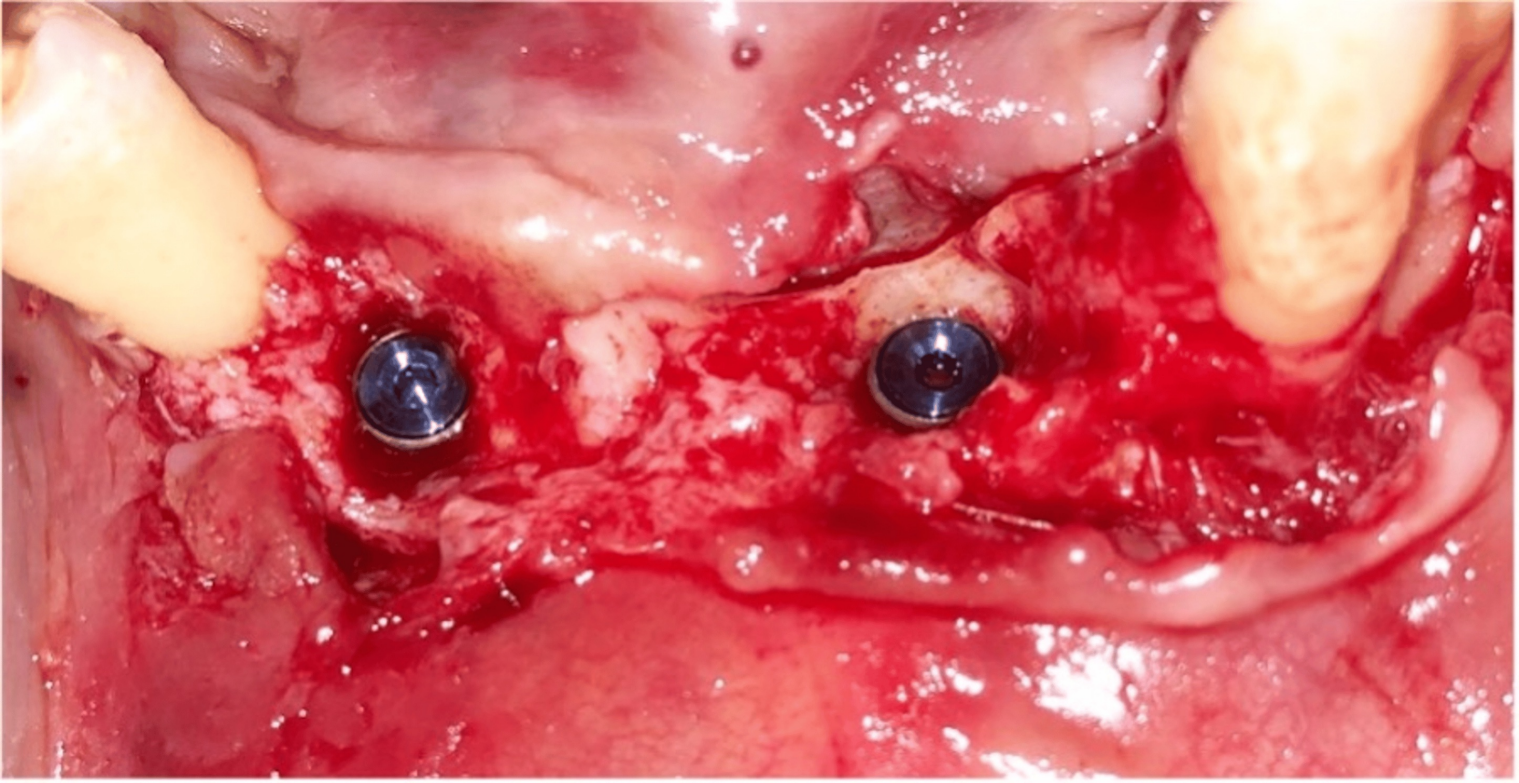
Cover-screws placed on the implants

**Figure 9:**
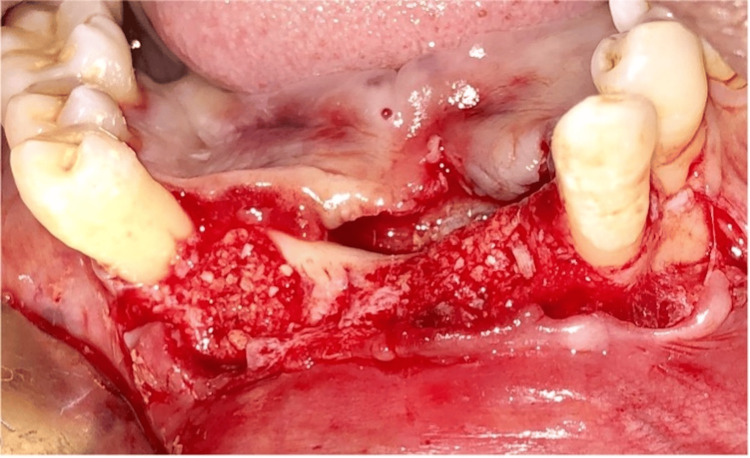
Bone grafting.

**Figure 10:**
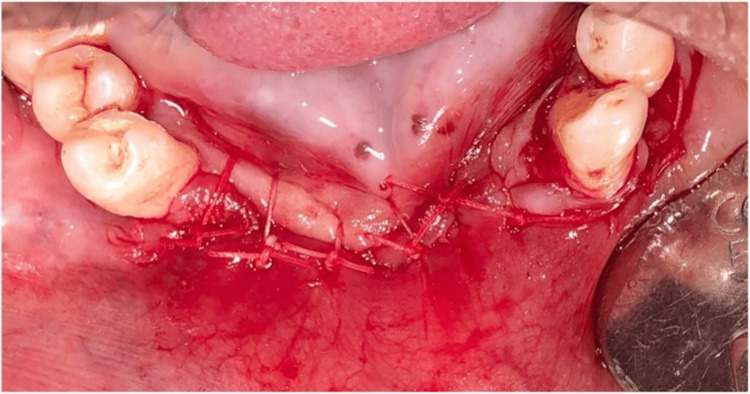
Sutures placed (PGA/PLA) PGA, polyglactin; PLA, polylactic acid

Pre-medication (amoxicillin 625 mg, deflazacort 6mg) was prescribed to the patient 24 hours before the procedure. On the day of surgery, rabeprazole, Diclofenac 50 mg, and serratiopeptidase 15mg were given one hour prior to the procedure. After the administration of local anesthesia, 4% Articaine with epinephrine 1:1,00,000 (Septanest, Septodont), atraumatic extraction of #43 and #31 was performed using luxators (Figures 2, 3). The flap was reflected (Figure 4), and implant (SpiralTM, AlphaBio Tec, Petah Tikva, Israel) of size 4.75*10 mm and 4.75*11.5 mm was placed (Figures 5, 6). Figure 7 shows the radiograph of the implants immediately after they were placed. Over the implants, the cover screws were placed (Figure 8). The freeze-dried bone allograft (Tata Memorial Hospital, Mumbai, Maharashtra, India) was placed on the defect site (Figure 9), and flaps were approximated with 3-0 polyglycolic acid suture (PGA)/polylactic acid (PLA) suture (Lotus Surgicals, Dehradun, Uttarakhand, India) (Figure 10).

**Figure 11:**
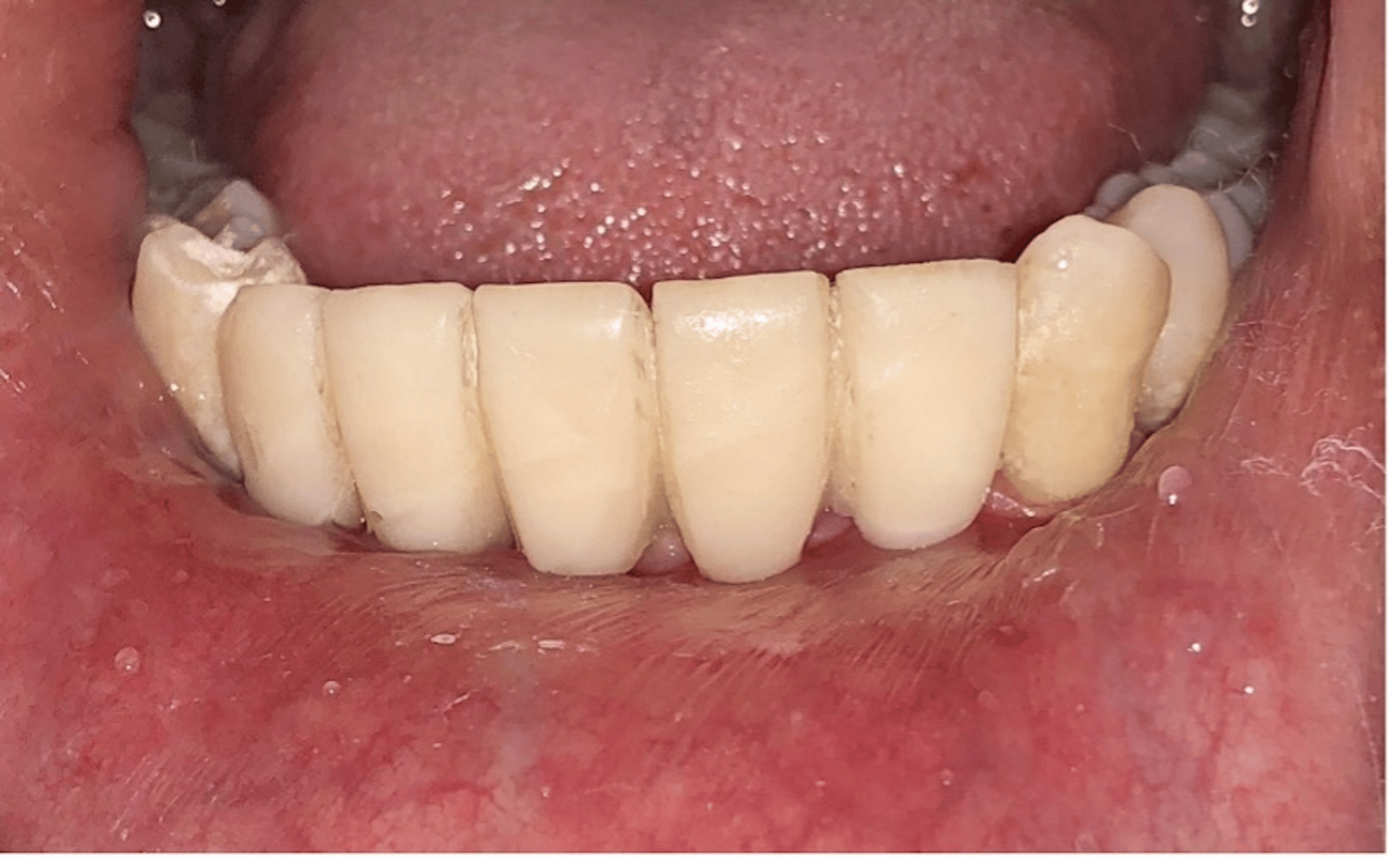
Maryland prosthesis for provisionalization

**Figure 12:**
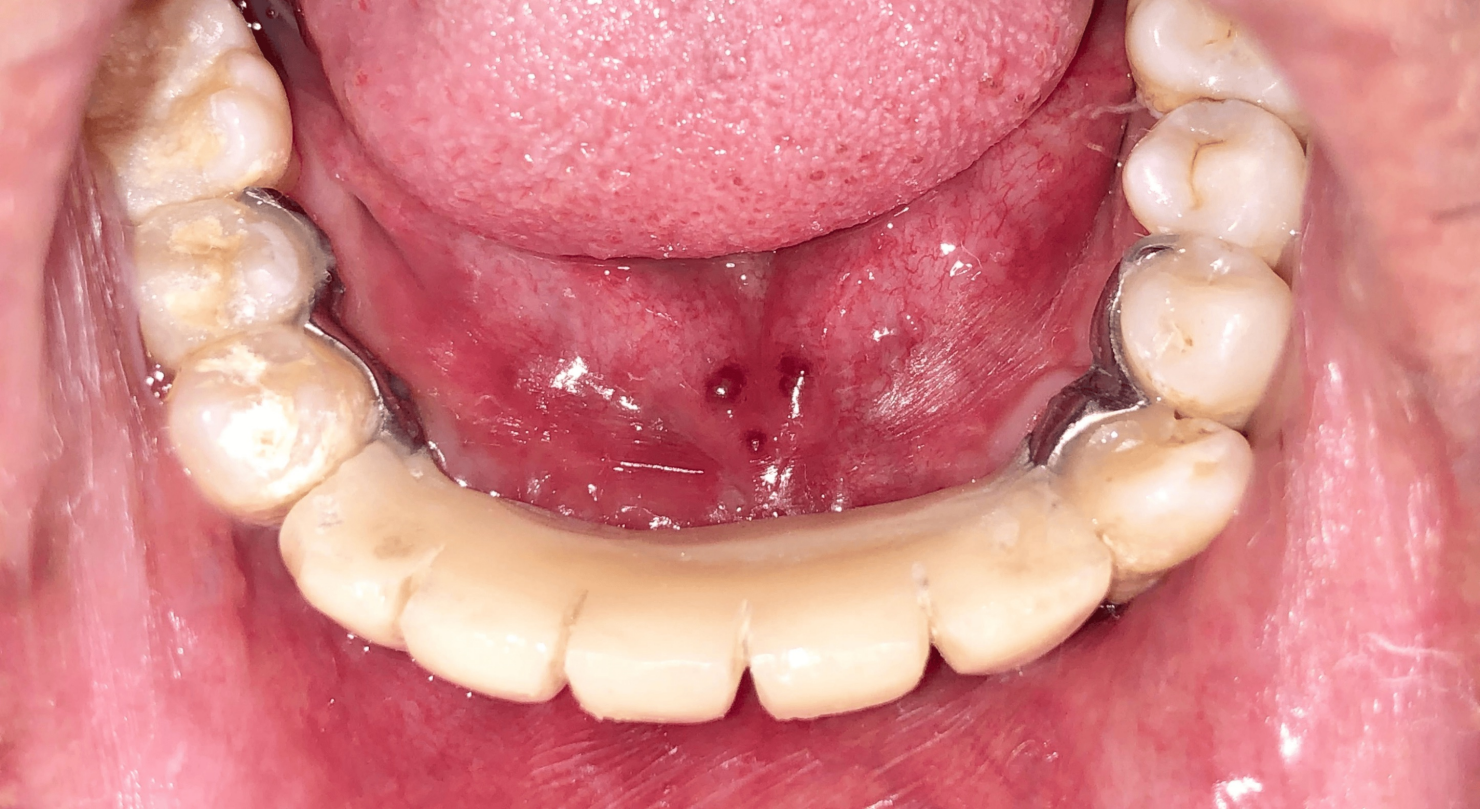
Lingual view of the Maryland prosthesis

A provisional Maryland prosthesis (Figures 11, 12) was provided after two weeks, and the patient was instructed to use a 0.12% chlorhexidine rinse twice daily for additional hygiene control.

Second-stage surgery

**Figure 13:**
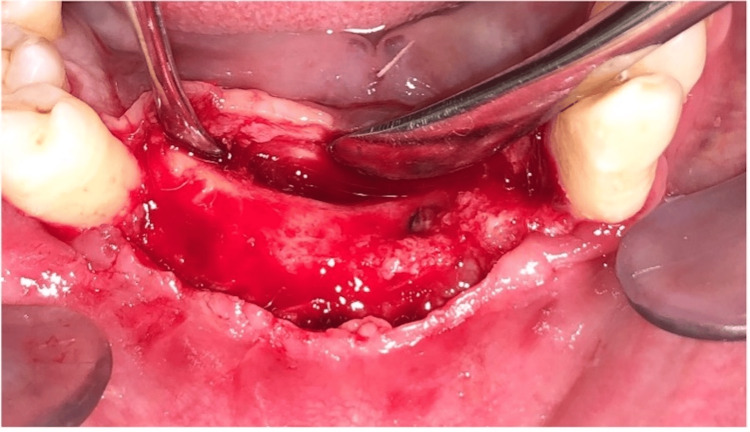
Second-stage surgery: mid-crestal incision

**Figure 14:**
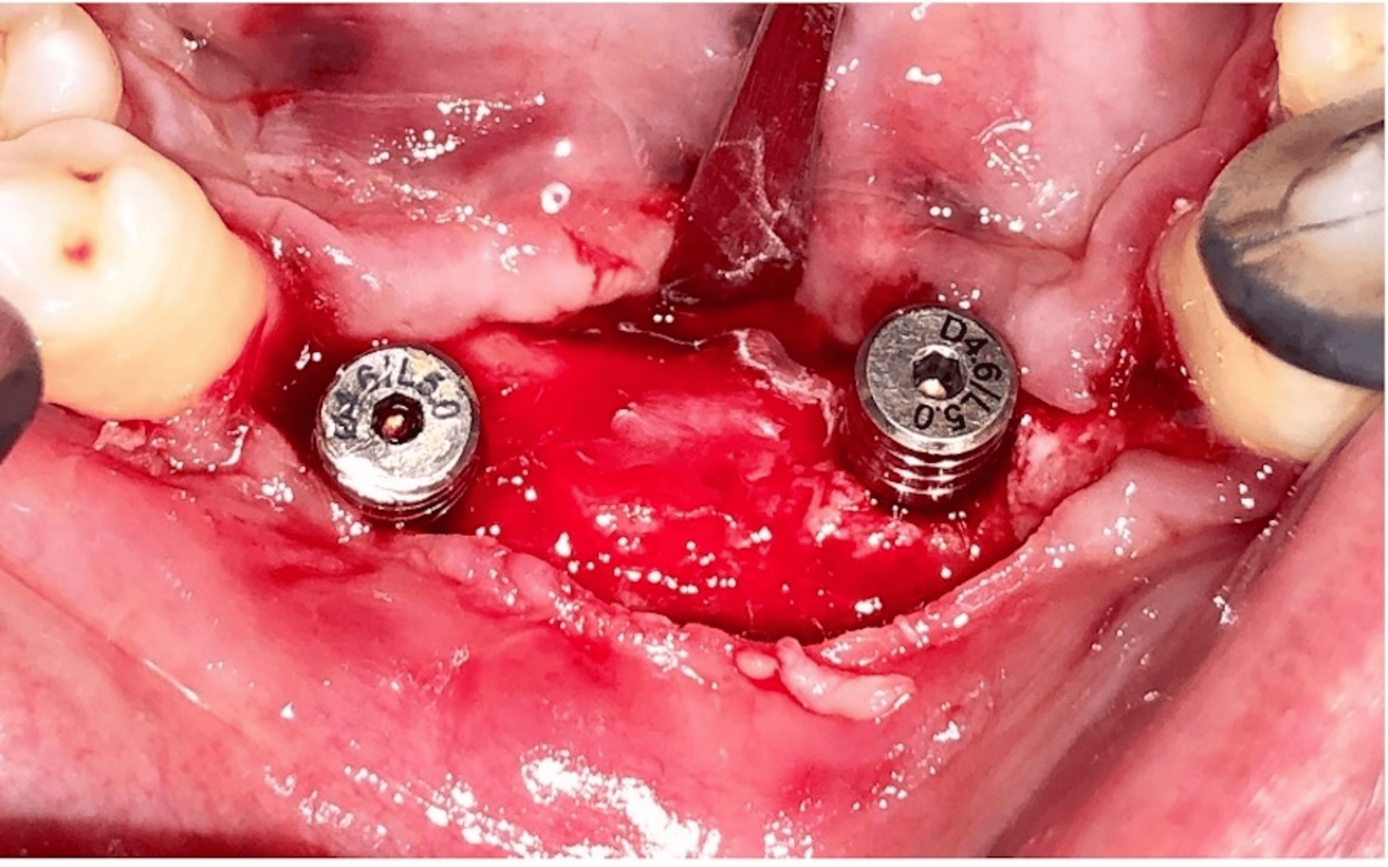
Gingival formers in place

**Figure 15:**
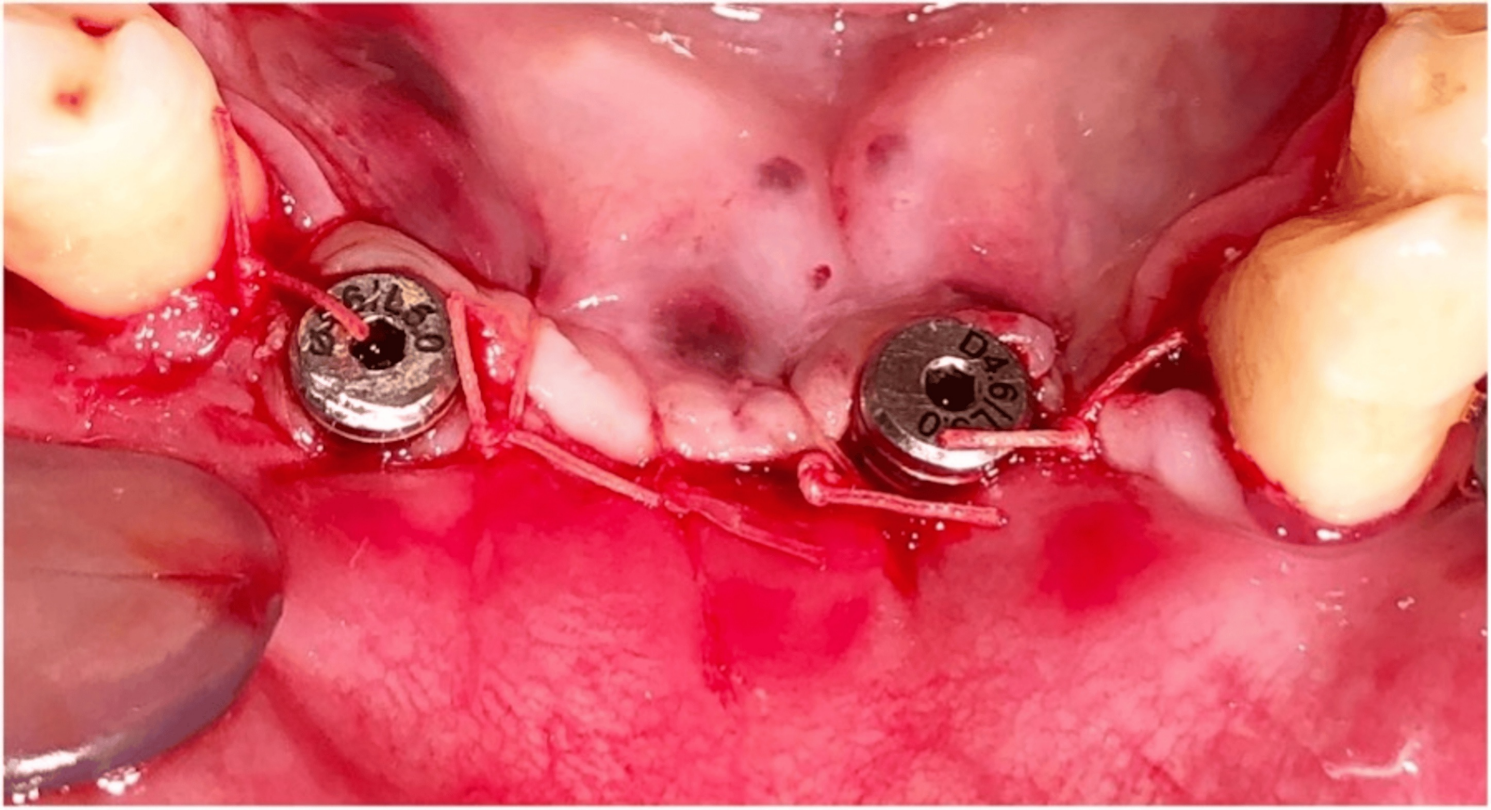
Suturing

The patient was recalled for second-stage surgery after five months. After clinical and radiographical evaluation, a mid-crestal incision (Figure 13) was made under local anesthesia, and after cover screws removal, healing abutments (gingival formers) were inserted (Figure 14) and the flap was approximated using PGA/PLA suture (Figure 15).

Prosthetic phase

**Figure 16:**
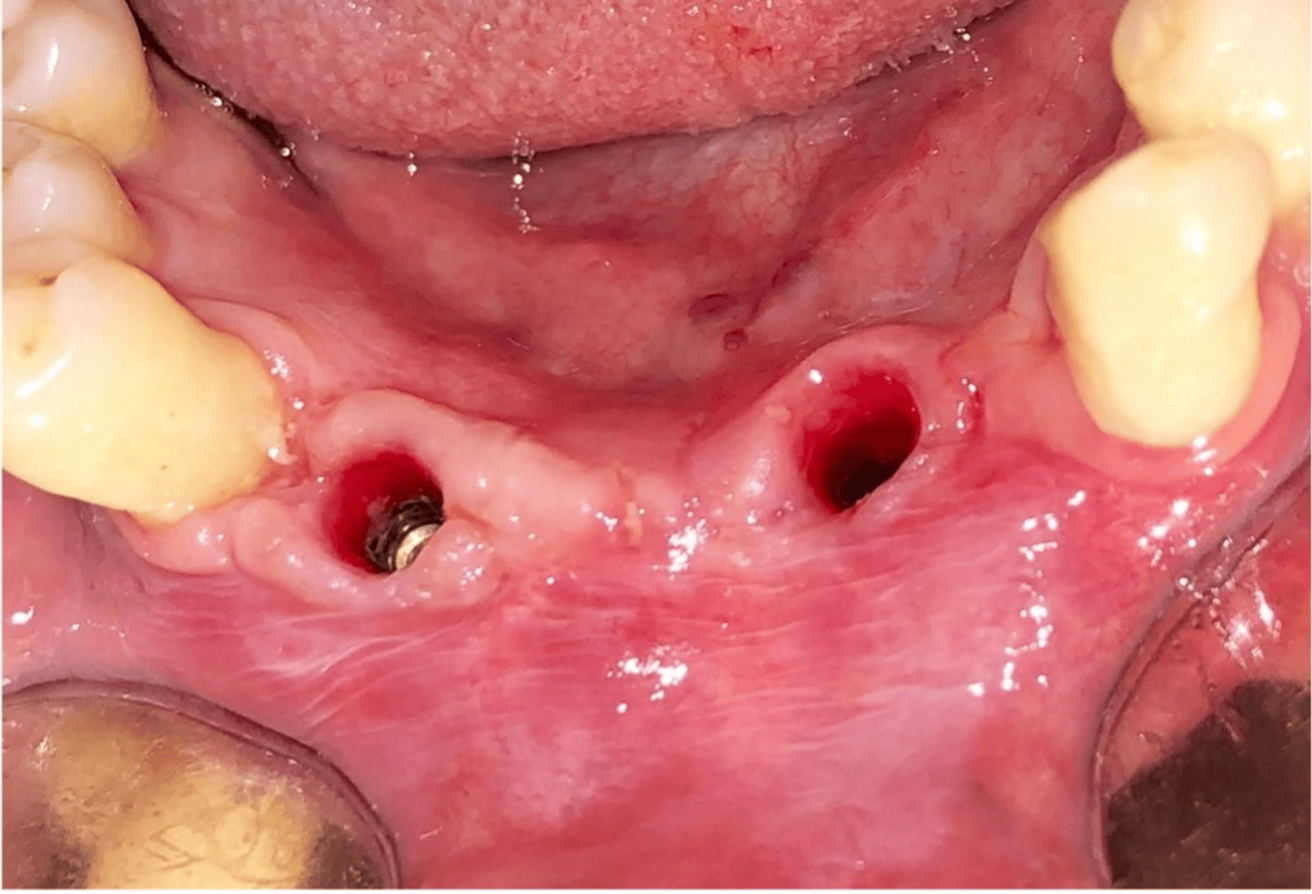
Gingival collar formation after two weeks

**Figure 17:**
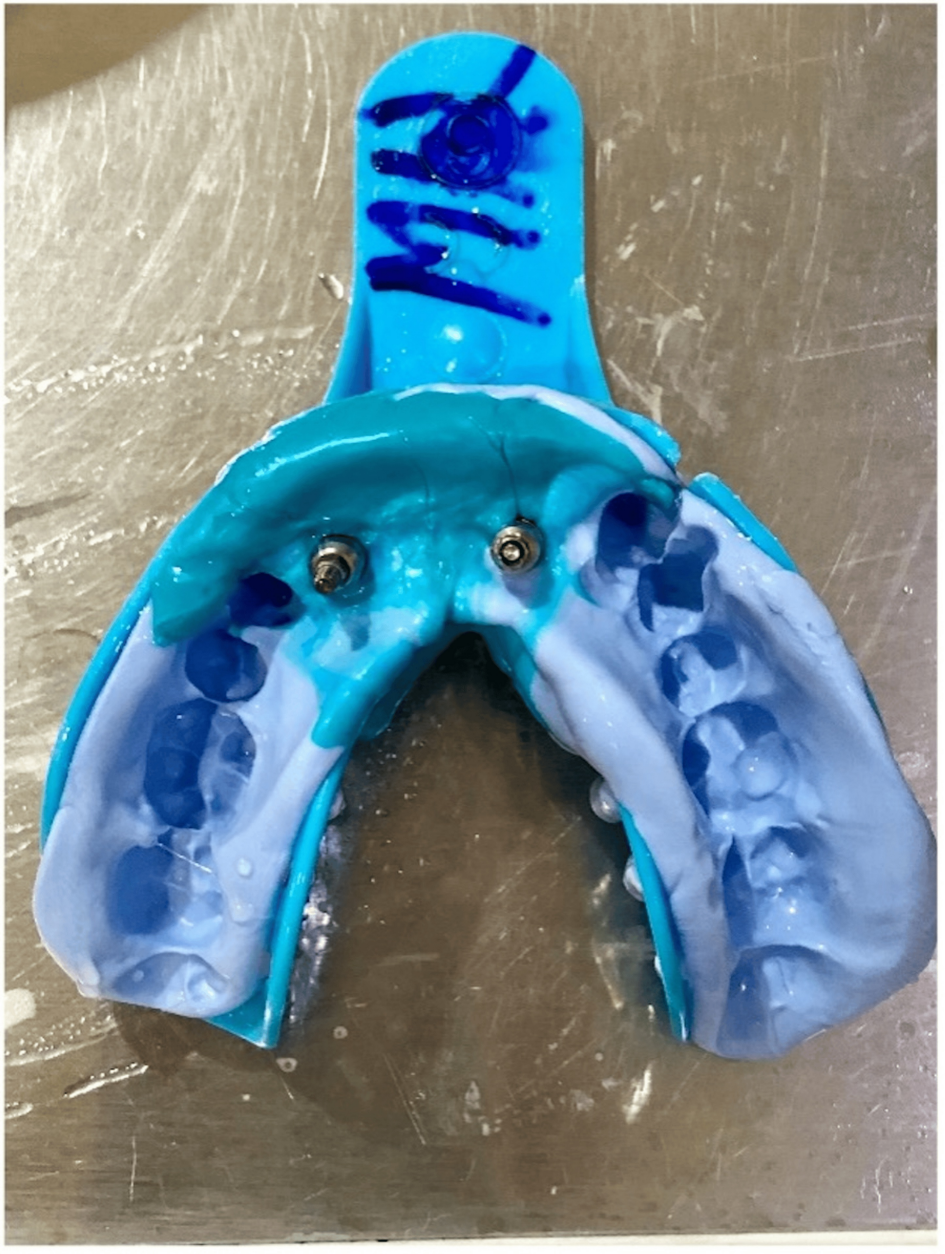
Impression made

**Figure 18:**
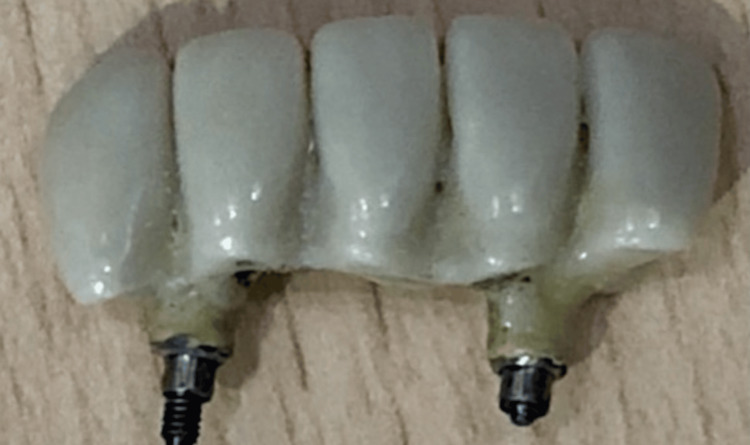
Prosthesis fabricated

**Figure 19:**
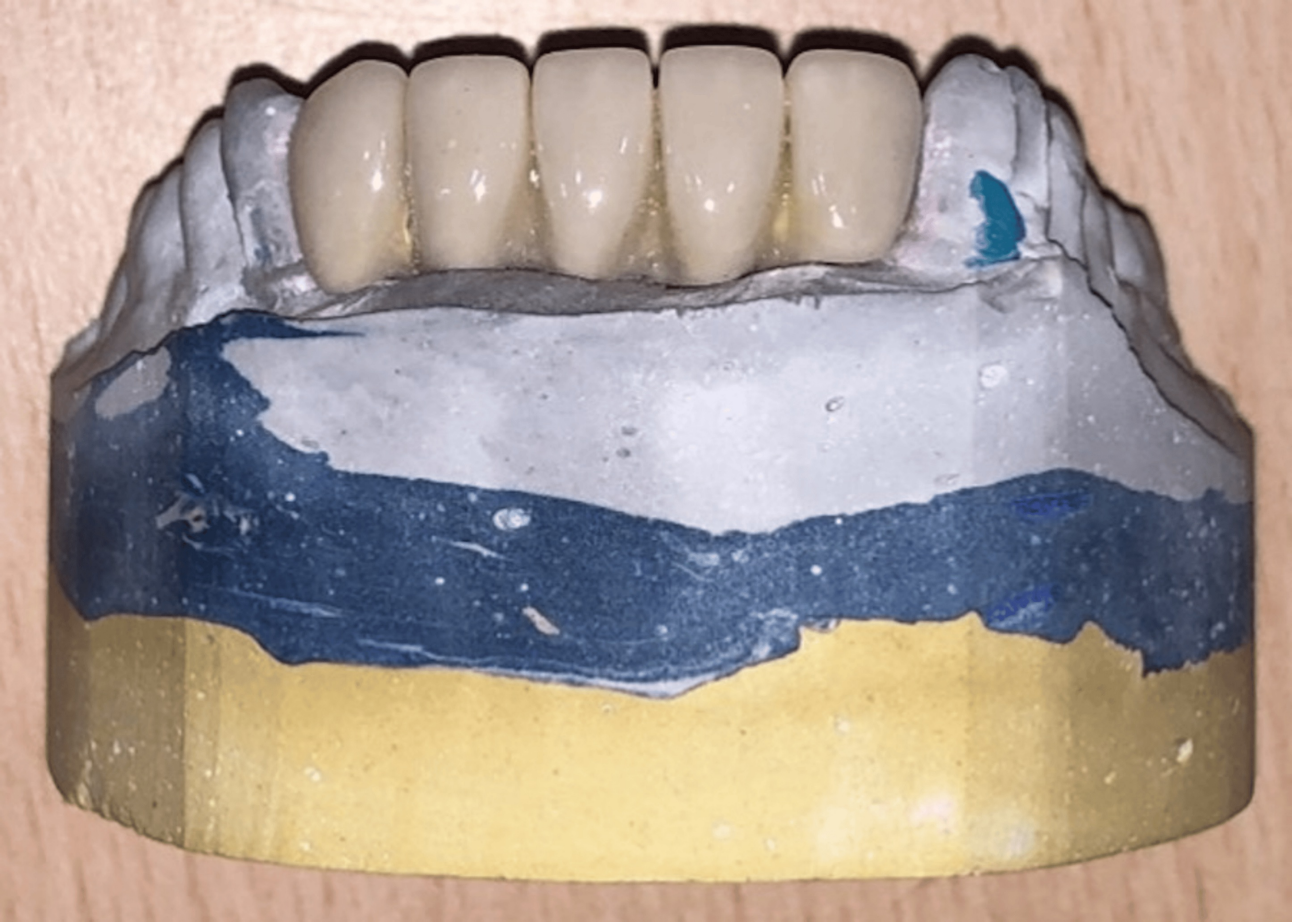
Try-in of the prosthesis on cast.

**Figure 20:**
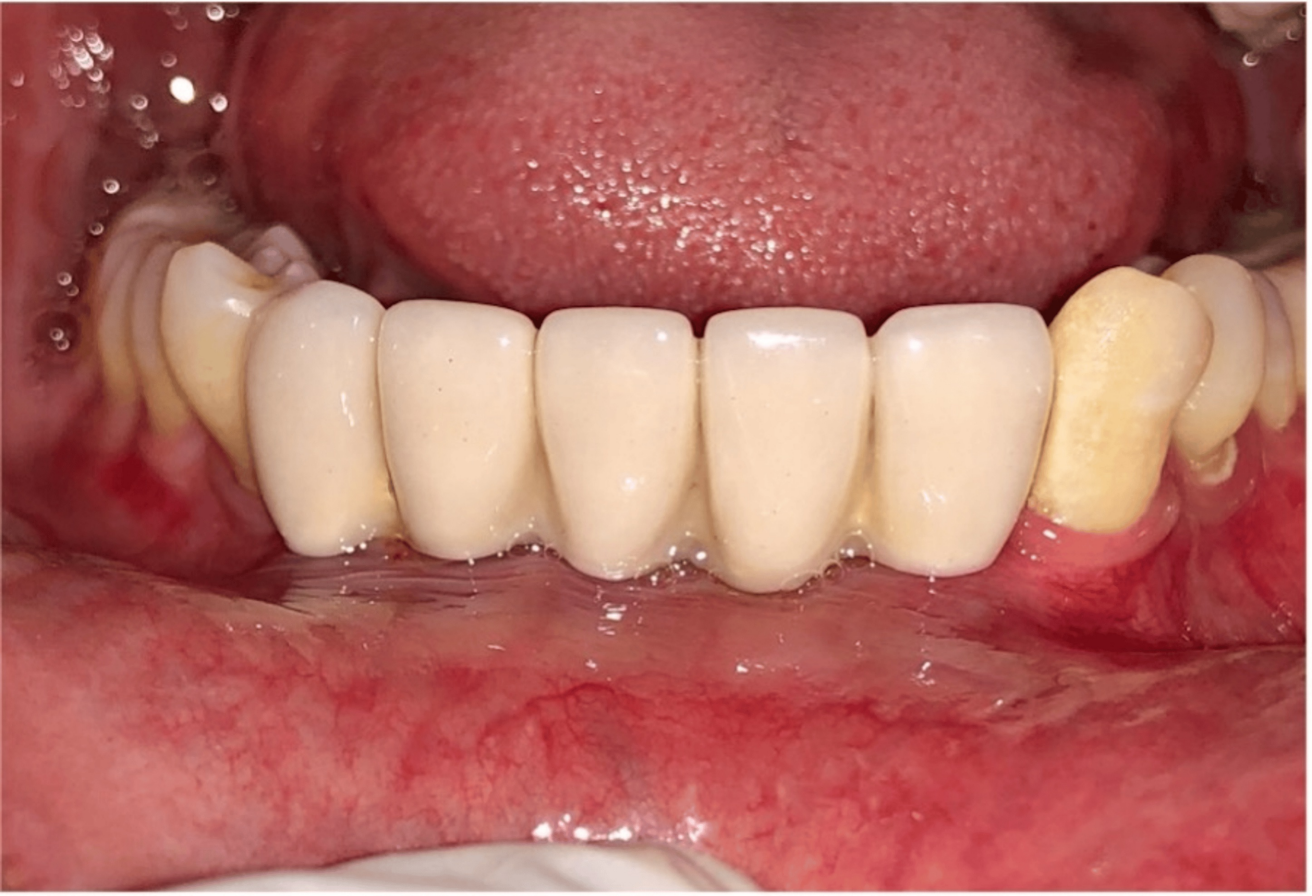
Final prosthesis (screws retained)

After two weeks of healing, the peri-implant collar, which forms around implants, was assessed (Figure 16). Impressions were made (Figure 17), and final screw-retained metal-ceramic prostheses were delivered (Figures 18-20). The patient was instructed to follow proper oral hygiene measures.

Follow-up and results

**Figure 21:**
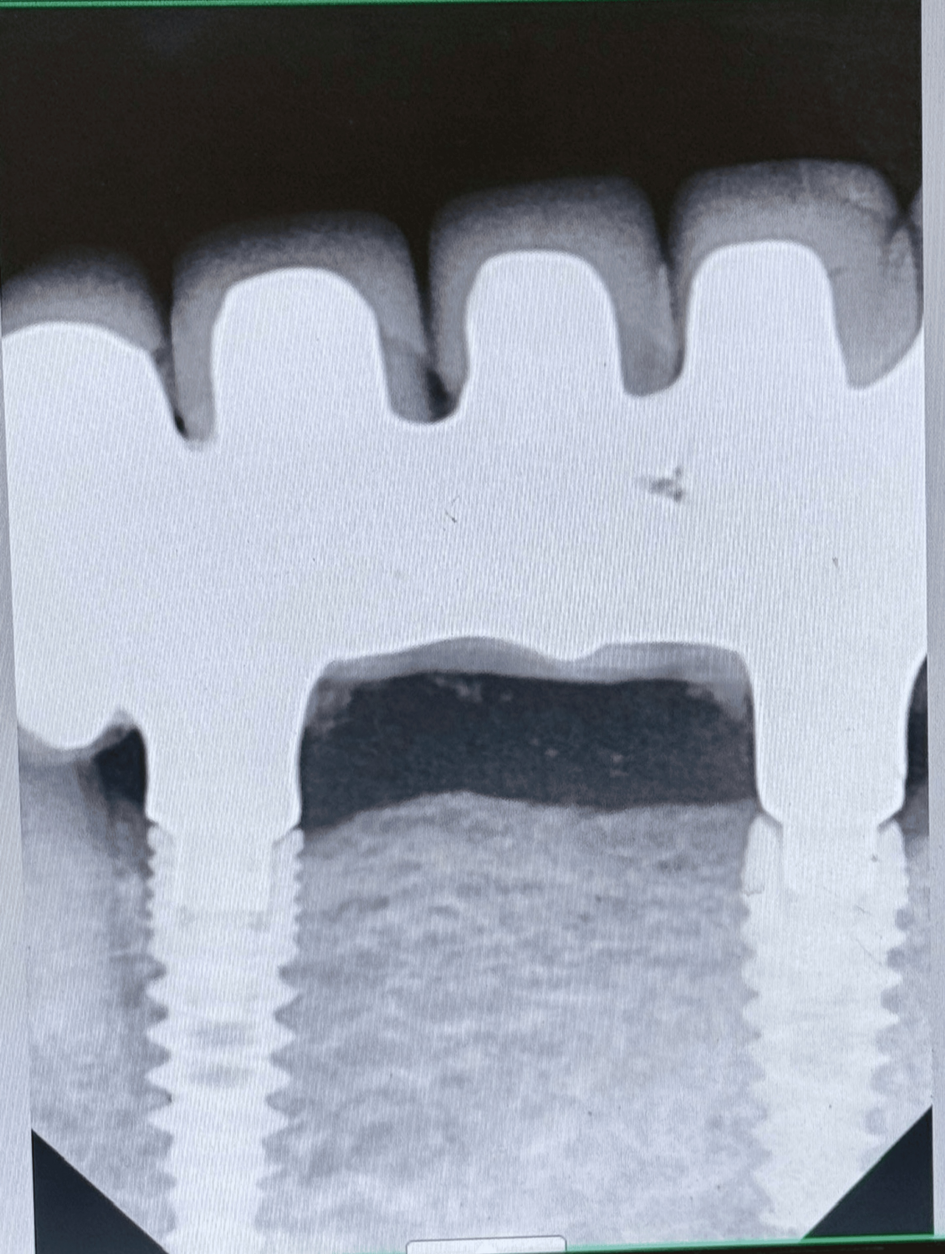
Radiovisiography of the implant site at the eight-year follow-up

**Figure 22:**
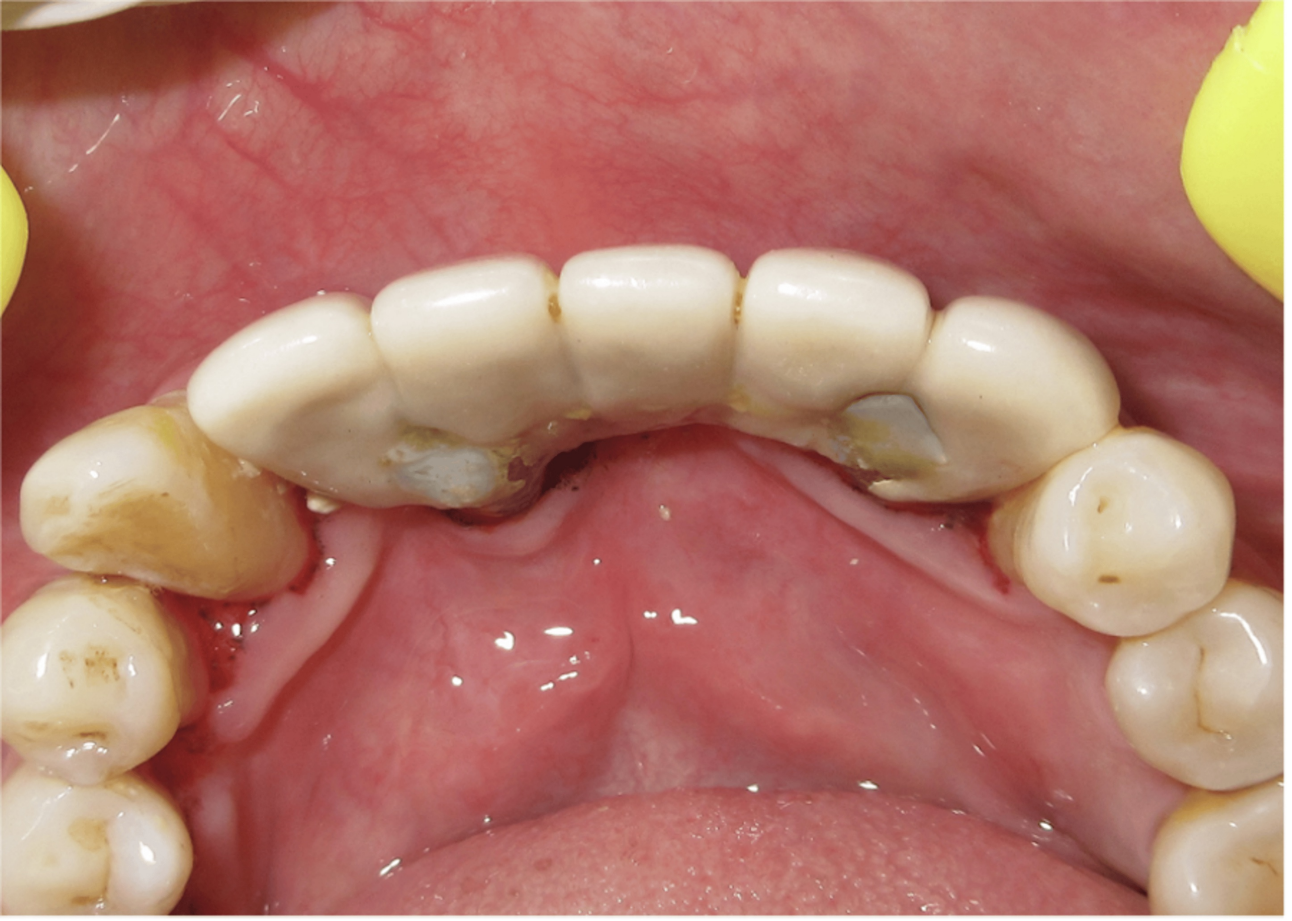
Lingual view at the eight-year follow-up

**Figure 23:**
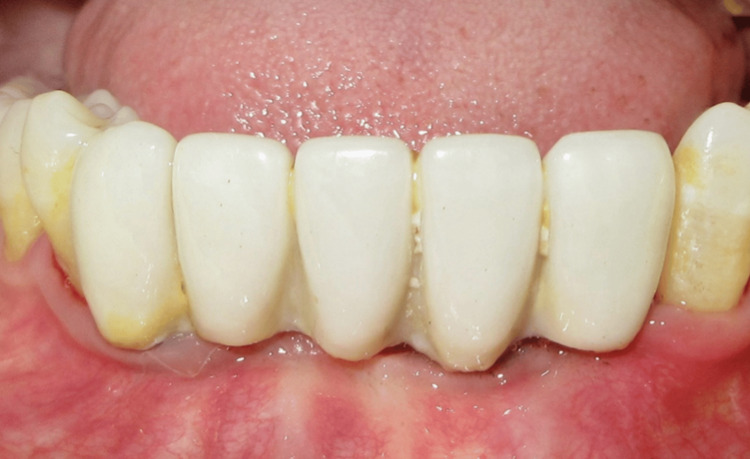
Facial view at the eight-year follow-up

Over the eight-year follow-up period, the patient showed stable periodontium and healthy implant with no sign of inflammation at the peri-implant area. The patient was closely monitored for clinical and radiographic changes, including full-mouth plaque score, bleeding index, probing pocket depth, and marginal bone loss (Figures 21-23).

At the eight-year follow-up, no implant loss was seen, and there were no complications such as ceramic fracture or abutment unscrewing. Radiographic analysis confirmed a healthy peri-implant environment, with bone levels stable within normal limits.

## Discussion

The outcomes of implant treatment in periodontally compromised patients have been widely debated. Our clinical report supports previous findings that while chronic periodontitis can complicate implant therapy, it does not necessarily prevent long-term implant success [9].

The patient in this study experienced stable implant survival and minimal complications over eight years of follow-up. A history of periodontitis is associated with increased risk for peri-implantitis and bone loss, but with proper periodontal treatment and maintenance, it can result in favorable outcomes [10]. Patients with a history of periodontitis had lower implant survival rates than patients without a history of periodontitis and are more prone to biological complications such as peri-implant mucositis and peri-implantitis [11]. This could be possibly due to factors such as microbial colonization and the inflammatory response in these patients [12].

Clinical outcomes of dental implant treatment in patients with generalized aggressive periodontitis for a follow-up period of more than five years were assessed, which showed a remarkable survival rate of 95.9% to almost 100% [13].

Our findings align with previous research indicating that while periodontitis may increase the risk of implant failure, a structured maintenance program can mitigate these risks. Regular follow-up visits, professional cleaning, and reinforcement of oral hygiene protocols are essential for ensuring long-term success in periodontally compromised patients [14].

Finally, in the present situation, though the patient has no keratinized gingiva, the patient was able to maintain oral hygiene. Thus, if a careful prophylaxis program is maintained, the position of the soft tissue remains static and stable [15].

Further research with a larger sample size and longer follow-up periods is needed to definitively conclude the long-term impact of periodontitis on implant survival.

## Conclusions

This clinical report demonstrates that dental implants can function successfully for more than eight years in patients with chronic periodontitis provided that proper periodontal treatment and maintenance are followed. Partially edentulous patients exhibit stable implant survival, suggesting that implants can be a viable option for individuals with chronic periodontitis when managed correctly. Regular periodontal maintenance and strict oral hygiene practices are essential to the long-term success of these implants.
